# Ectopic Tooth in the Roof of the Left Maxillary Sinus

**DOI:** 10.7759/cureus.49765

**Published:** 2023-12-01

**Authors:** Isabel Vazquez, Mariana Cebotari, Flávia Pereira, Lígia Coelho, Teresa Burnay

**Affiliations:** 1 Maxillofacial Surgery Department, Centro Hospitalar Universitário de São João, Porto, PRT

**Keywords:** chronic sinusitis, maxillofacial surgery, dental surgery, caldwell-luc approach, maxillary sinus, sinusitis, ectopic tooth

## Abstract

The presence of sinonasal ectopic teeth is a rare entity that is usually asymptomatic. In some cases, the presence of foreign bodies in the maxillary sinus, such as ectopic teeth, can lead to chronic maxillary sinusitis. We report a case of chronic sinusitis because of an ectopic tooth in the roof of the left maxillary sinus in a 50-year-old female who presented with complaints of facial pain in the left maxillary region and purulent nasal discharge. The treatment of ectopic teeth usually consists of the removal of the previous, taking into account its location and possible risks. In this case, the close proximity to the orbit could have led to a greater risk of complications involving the infraorbital bundle. CT scan evaluation is frequently required to identify the exact location and is useful for treatment planning. The traditional surgical approaches to maxillary sinus pathology are transoral Caldwell-Luc approaches or transnasal endoscopic surgery. The method used in this case was the Caldwell-Luc approach. Although more invasive, it allows visualization into the maxillary sinus and superior access for instrumentation of the posterolateral region while permitting manipulation and removal of larger objects. Despite maxillary sinus ectopic teeth being uncommon, it is important for clinicians to become aware and to consider this entity to provide early adequate treatment.

## Introduction

Ectopic teeth can be described as teeth that develop and/or erupt away from their natural anatomic position and can be found in 1% of the total population [1-3]. The third molars are the teeth mostly found in ectopic locations, and the lower jaw is affected more frequently than the maxilla [4-5].

In the maxillofacial area, these have been described in the mandibular condyle, coronoid process, mentum, maxillary sinus, nose, and orbit [6]. Even though the causes for this entity remain unclear, it is believed to occur secondary to trauma, iatrogenic procedures, infection, crowding, dense bone, and developmental disorders [4,7,8]. However, in many cases, the etiology is not determined [5,8].

The presence of sinonasal ectopic teeth is a rare entity that is usually asymptomatic [4,9]. On a few occasions, chronic maxillary sinusitis because of the presence of foreign bodies in the maxillary sinus, such as ectopic teeth, have been described [8]. The most common cause for maxillary sinus foreign body is a history of dental procedures such as dental implant placement or third molar extraction with iatrogenic sinusal migration [8].

This paper aims to present a case of an ectopic third molar tooth in the roof of the left maxillary sinus and its management.

## Case presentation

A 50-year-old female was referred to the Department of Maxillofacial Surgery because of facial pain in the left maxillary region, with six months of evolution, accompanied by local infections with purulent nasal discharge. There were no significant diseases, history of trauma, or relevant dental procedures in the patient’s anamnesis. Physical intraoral examination revealed, in the second quadrant, the absence of first and third molars, with no other significant findings. The left superior first molar had been previously extracted, and the third molar never erupted.

**Figure 1:**
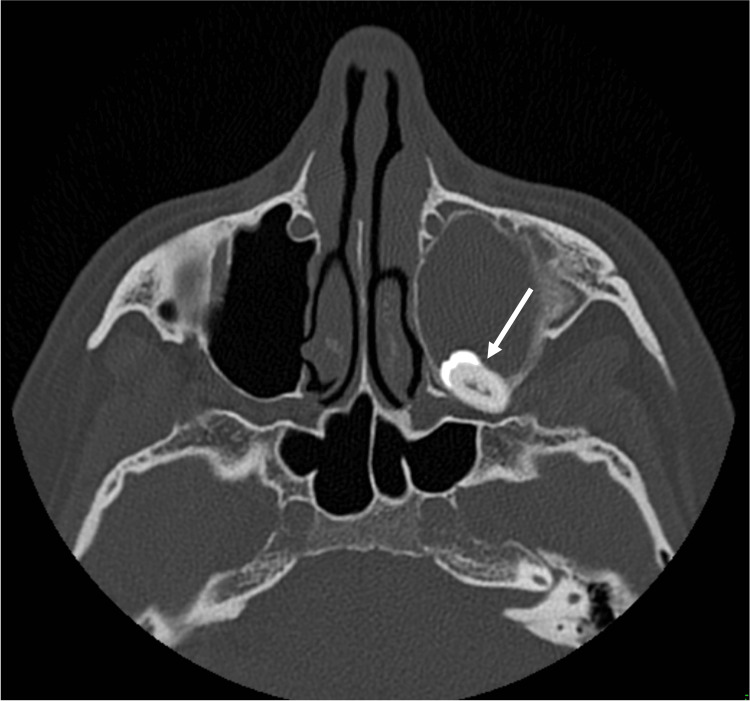
Preoperative maxillofacial CT scan Axial view of preoperative maxillofacial CT scan showing an isodense area with complete obliteration of left maxillary sinus and an ectopic tooth (arrow) in the posterior portion of the roof of the sinus

**Figure 2:**
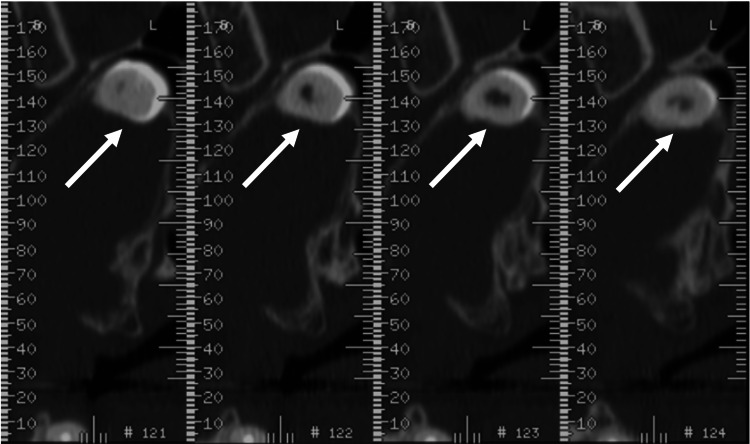
Preoperative dentascan Dentascan cross-sectional views of the maxilla showing an ectopic tooth (arrows) in the roof of the left maxillary sinus and a fluid-filled sinus

**Figure 3:**
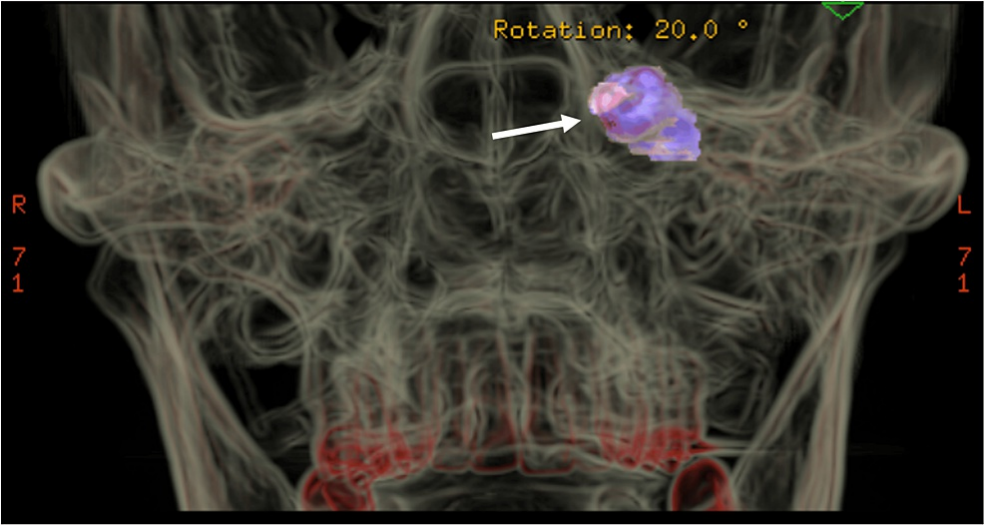
Three-dimensional reconstruction of preoperative maxillofacial CT scan Three-dimensional view of CT scan showing a highlighted ectopic tooth encroaching the posterosuperior wall of the left maxillary sinus

Panoramic radiograph and CT scan imaging indicated the presence of an ectopic tooth with a total length of 15 mm in the posterior portion of the roof of the left maxillary sinus, in close contact with the infraorbital vessels and nerve and optic canal, and a fluid-filled sinus (Figures 1-3).

**Figure 4:**
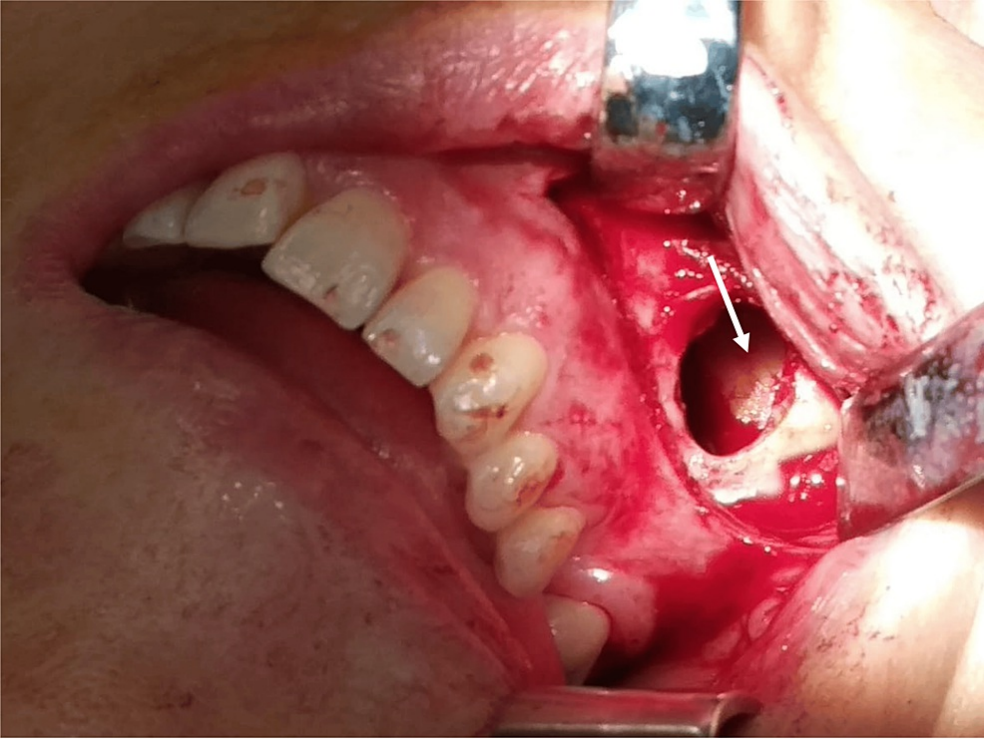
Intraoperative image of surgical approach and ectopic tooth Intraoperative image of Caldwell-Luc approach showing vestibular incision, osteotomy in the premolar region, and ectopic tooth in the maxillary sinus (arrow)

The ectopic tooth extraction was planned. A transoral Caldwell-Luc approach was performed with a vestibular incision in the second quadrant, followed by the osteotomy of the anterior maxillary wall in the premolar region, creating a bony window, which was removed and preserved. The interior of the maxillary sinus was filled with purulent content, which was drained (Figure 4).

**Figure 5:**
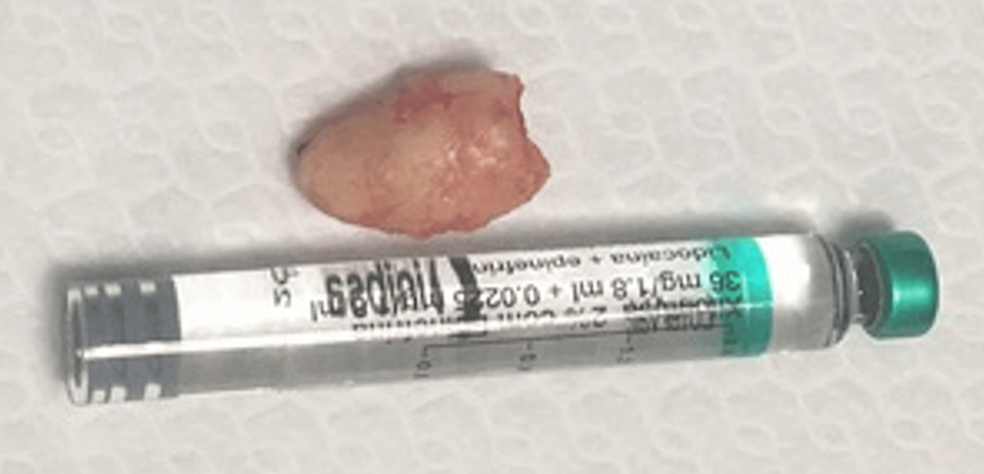
Extracted ectopic tooth Ectopic tooth removed from the left maxillary sinus

After identification of the ectopic tooth, which was partially covered by a thin bone layer, careful luxation with a dental elevator and odontectomy were performed (Figure 5).

Subsequently, curettage of the affected Schneiderian membrane, middle meatal antrostomy, and closure of the bony defect with a repositioning of the bony window were executed. Antibiotic treatment with amoxicillin-clavulanate 875+125 mg twice daily was administered for seven days. In the postoperative period, the patient presented favorable evolution. Histopathological examination of the collected Schneiderian membrane revealed features suggestive of chronic sinusitis. The patient was clinically and radiographically evaluated for one year after surgery with no signs of sinusitis recurrence.

## Discussion

Although the etiology of ectopic teeth remains undetermined, it is considered that it may be related to abnormal interactions concerning the oral epithelium and mesenchymal tissue during odontogenesis [5,7]. These cases are usually incidental findings in routine dental radiographic evaluations [4,7]. Radiographically, ectopic teeth appear as radiopaque lesions with the same attenuation as that of the oral teeth, with a central radiolucency related to the pulp cavity [6].

The presence of ectopic teeth in the maxillary sinus is most frequently asymptomatic [7,10]. When symptomatic, it can present as numbness of the maxillary area, facial pain, edema, sinusitis, headache, epiphora and/or oroantral fistula, and all of which lack specificity [1,7,11].

One of the most common causes of paranasal sinus ectopic teeth is dental trauma; therefore, dental treatment history is essential [8]. In our case, no history of trauma, invasive dental procedure, or pathological condition was reported.

Ectopic teeth found in the maxillary sinus may be deciduous, permanent, or supernumerary, and most commonly are ectopic third molars [4,5,7]. It is thought that sinusal foreign bodies lead to chronic sinusitis because of constant mucosal irritation that prompts infection and ciliary insufficiency [8]. Therefore, it is expected to resolve after foreign body removal and antibiotic administration [8]. 

In many cases, a CT scan evaluation is required to identify the exact location and is useful for treatment planning [7]. In cases related to chronic sinusitis, frequent CT findings are the thickening of the mucosa and opacification of the sinus, along with the ectopic tooth [8].

The treatment of ectopic teeth usually comprises removal of the previous, taking into account its location and possible risks [4]. The traditional surgical approaches to maxillary sinus pathology are transoral Caldwell-Luc approaches or transnasal endoscopic surgery [5,12]. In recent literature, there is shift towards transnasal, endoscopic management of this pathology through a middle meatal antrostomy. This method is less invasive compared to open surgery and is usually associated with shortened operative times, less perioperative morbidity, and reduced hospital length of stay. However, its usage is limited in cases of severe nasal obstruction, severe epistaxis, craniofacial trauma with a risk of inadvertent intracranial instrumentation, or difficult tooth position. To achieve retrieval of a maxillary sinus tooth using this approach, it is required to open widely the medial wall of the maxillary sinus [8].

Comparing these techniques, even though the Caldwell-Luc approach is more invasive and requires greater bone removal, it provides direct visualization into the maxillary sinus and superior access for instrumentation of the posterolateral region of the previous, while allowing manipulation and removal of larger objects during surgery [12]. To avoid a big bone defect, the repositioning of the bony window is an alternative method to restore the integrity of the anterior wall of the maxillary sinus. 

In this case, the close proximity to the orbital floor could have led to a greater risk of complications involving the infraorbital vases and nerve, such as intraoperative hemorrhage and postoperative numbness of the ipsilateral upper teeth and the skin of the cheek, upper lip, and lateral aspect of the nose. However, the procedure was uneventful.

## Conclusions

Sinonasal ectopic tooth is a rare entity, and the treatment of choice is surgical removal as it can lead to greater complications if left untreated.

This case illustrates the occurrence of chronic sinusitis because of the presence of an ectopic tooth in the roof of the left maxillary sinus. The surgical removal of the ectopic tooth through a Caldwell-Luc approach led to the resolution of the symptoms with an uneventful recovery. Even though there are growing reports of endoscopic surgery to operate on sinonasal ectopic teeth, classic surgery methods such as the Caldwell-Luc approach should be kept in mind as it provides superior access for instrumentation of the posterolateral region of the maxillary sinus and allows manipulation and removal of larger objects during surgery. Therefore, while maxillary sinus ectopic teeth are uncommon, it is important for clinicians to be aware of this entity, provide early treatment and be mindful of classic surgery approaches that can be advantageous in complex cases.
